# Distress and Well-Being Among Psychiatric Patients in the Aftermath of the First COVID-19 Lockdown in Israel: A Longitudinal Study

**DOI:** 10.3389/ijph.2022.1604326

**Published:** 2022-06-01

**Authors:** Ariella Grossman-Giron, Dana Tzur Bitan, Shlomo Mendlovic, Sharon Shemesh, Yuval Bloch

**Affiliations:** ^1^ Department of Behavioral Sciences, Ariel University, Ariel, Israel; ^2^ Shalvata Mental Health Center, Hod HaSharon, Israel

**Keywords:** COVID-19, well-being, distress, psychiatric patients, public mental health

## Abstract

**Objectives:** Studies assessing the effect of the COVID-19 pandemic on psychiatric patients have mostly focused on cross-sectional evaluations of differences in levels of distress. In this study, we aimed to assess changes in distress and well-being following the COVID-19 pandemic outbreak as compared with pre-pandemic levels, as well as potential predictors of symptomatic deterioration, among psychiatric outpatients treated in a public mental health hospital in Israel.

**Methods:** Patients evaluated for distress and well-being before the pandemic (*n* = 55) were re-evaluated at the end of the first lockdown in Israel.

**Results:** Analyses revealed a significant decrease in the patients’ sense of personal growth. Increases in distress were significantly associated with fear of COVID-19 beyond patient characteristics.

**Conclusion:** These results suggest that the pandemic has a short-term effect on patients’ well-being, and that fear of the pandemic is associated with elevations in distress.

## Introduction

The COVID-19 outbreak has led mental health clinicians and researchers to express concerns regarding the psychosocial adverse consequences of the pandemic, also referred to as the “second pandemic” of mental health crises [1, 2]. Several factors have been proposed to facilitate the deterioration in public mental health, such as the high levels of fear arising from this infectious disease, prolonged periods of quarantine, and the need for social distancing [3, 4]. Studies assessing the overall level of distress among the general population provide some evidence for these predictions. For example, in a cross-sectional survey conducted in China, the overall prevalence of generalized anxiety disorder was 35.1%, and the prevalence of reported depressive symptoms and poor sleep quality were 20.1% and 18.2%, respectively [5]. In Italy, researchers reported increased rates of distress levels, as well as higher scores of depression, anxiety, and stress, as compared with a normative sample [6]. These findings support clinicians’ concerns regarding the possible mental health consequences of COVID-19.

One of the populations most susceptible to the mental health effects of the current pandemic is the population of psychiatric patients. Previous studies assessing the response of psychiatric patients to the pandemic have thus far focused on the comparison between clinical and normative populations, showing a trend of higher distress among the psychiatric group. For example, Hao et al. [7] assessed the immediate psychological impact of the COVID-19 lockdown in China among 76 psychiatric patients and 109 healthy controls, and found significantly higher levels of anxiety, depression, stress, insomnia, PTSD symptoms, and suicidal ideation among the psychiatric population. Iasevoli et al. [8] reported that psychiatric patients were four times more likely to express high COVID-19 pandemic-related stress, as compared to healthy participants, after 1 month of lockdown in Italy. In Australia, individuals with a self-reported history of mental illness exhibited higher levels of fear of COVID-19, health-related anxiety, and contamination fears than those without pre-existing diagnoses [9]. These results suggest that psychiatric patients might be more vulnerable to the effects of the pandemic compared to the general population. Nonetheless, changes in distress and well-being occurring during the pandemic, as well as potential predictors of them, have not been sufficiently delineated.

Studies assessing changes in distress among psychiatric patients before and after the outbreak of the pandemic are scarce. Nonetheless, scholars have previously suggested that loneliness surrounding the pandemic, as well as impairments to daily routine and loss of positive activities, can adversely impact preexisting mental health disorders [10]. Yao et al. [11] further proposed that the strict lockdown and restrictions may have prevented psychiatric patients from help-seeking, which may have added to their general distress. Studies assessing the response of psychiatric patients to major life events have previously demonstrated that psychiatric patients are vulnerable to psychological distress reactions following the event. For example, in a naturalistic longitudinal study conducted in the US, the authors found that approximately 20% of psychiatric patients with bipolar disorder experienced a new onset of PTSD symptoms following indirect exposure to the 9/11 terrorist attacks [12]. Thus, patients suffering from mental distress might exhibit negative psychological effects after a major stressful event, such as the outbreak of the COVID-19 pandemic.

In this study we aimed to assess the psychological consequences of COVID-19 for the psychiatric population, by conducting a follow-up examination among outpatients struggling with severe mental illnesses. To reach this aim, we reapproached psychiatric patients who reported on various mental health facets prior to the pandemic as part of a previous study [13]. Patients who agreed to participate in the current study were re-evaluated for symptomatic distress, well-being, and fear of COVID-19 right after the lifting of the first lockdown in Israel (mid-May 2020). Based on past literature pointing to the negative mental health effects of the current pandemic [7, 8], it was hypothesized that patients would exhibit elevations in distress, as well as a decrease in well-being after the pandemic’s outbreak. Furthermore, it was hypothesized that fear of the pandemic would be positively associated with elevation in distress.

## Methods

### Setting

The study was approved by the Shalvata Mental Health Center institutional review board (IRB, approval number: 0007-20-SHA). Patients who had participated in a previous study which was carried out in October 2017 [13] were contacted after the lifting of the first lockdown in Israel, at mid-May 2020. Baseline measures of level of distress before the pandemic were extracted from the last measurement of the previous study. All patients were diagnosed in October 2017 using an unstructured clinical interview. This interview was performed by a senior psychiatrist during a staff intake meeting in each unit of the psychiatric hospital. The mean time gap between the first measurement (obtained from the previous trial) and the second measurement (collected at the present time point) was 30.85 months (SD = 4.69). Re-evaluation of the patients’ status after the lockdown was performed via an online survey. Patients signed an online informed consent form prior to the completion of the survey.

### Participants

**FIGURE 1:**
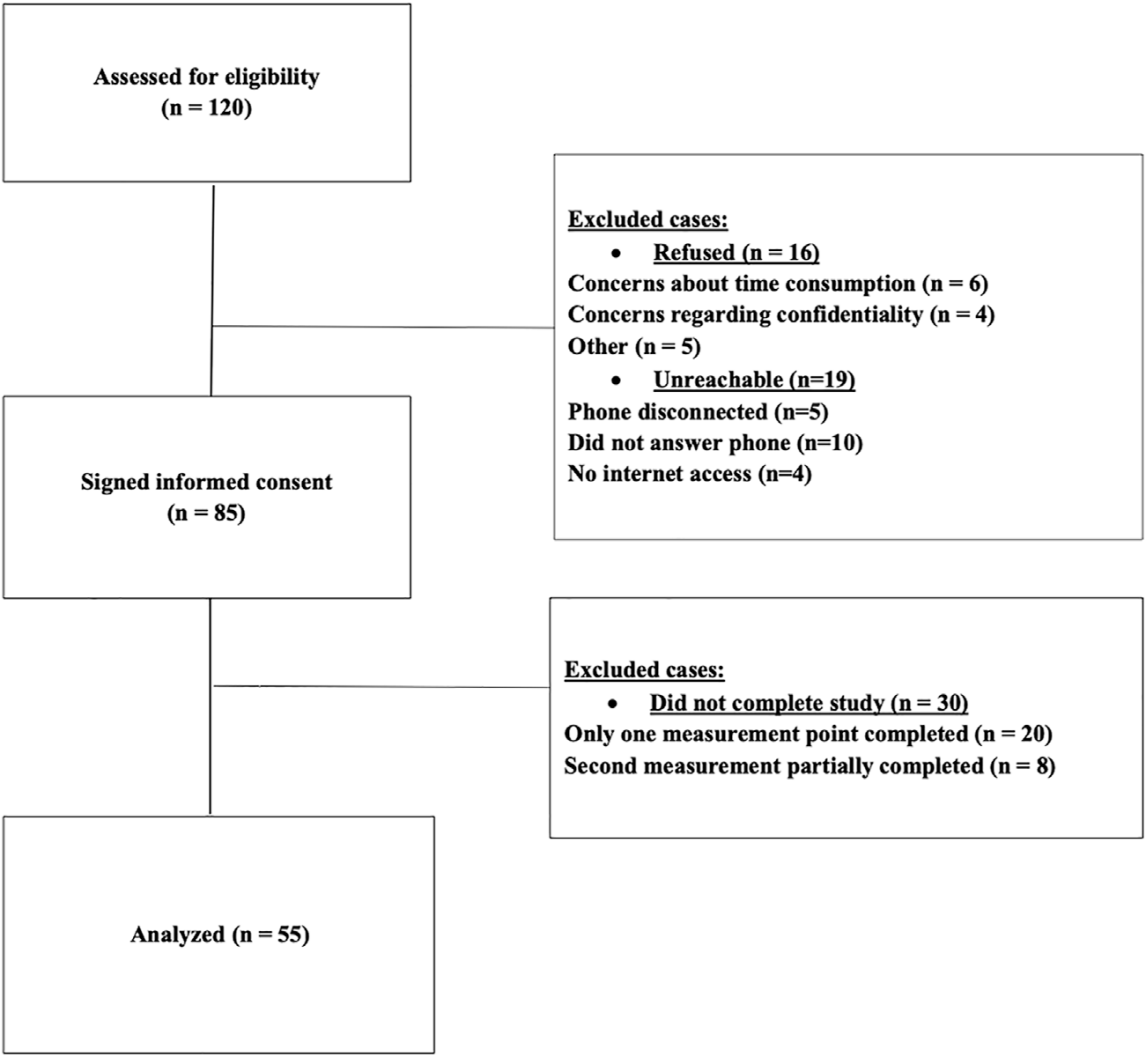
Flowchart of patients’ enrollment. Distress and well-being among psychiatric patients in the aftermath of the first COVID-19 lockdown in Israel: A longitudinal study, Israel, 2020.

Adult outpatients from four units of Shalvata MHC, who had participated and completed measurements in the previous study, were assessed for eligibility for the current study. Inclusion criteria included the following: provision of informed consent; adequate understanding of the Hebrew language; participating individual or group therapy as part of the previous study; and completion of at least one measurement at baseline. A total of 120 participants were approached. Of these potential participants, 19 were either unreachable (e.g., did not answer their phone) or unable to obtain access to the online survey due to lack of internet service, while 16 declined to participate. Common reasons for refusal included concerns about the survey being time-consuming and apprehensions about confidentiality. Of the total 85 patients who gave their consent to participate in the current study, 30 did not fully complete the survey. For the purposes of the current study, we only analyzed patients with two complete measurement points, resulting in a total sample of 55 participants. Figure 1 presents a flowchart of the enrollment process of the current study’s participants.

### Measures

The Outcome Questionnaire-45 (OQ-45) [14]. A self-report questionnaire devised to assess patient outcomes over the course of therapy. This measure consists of 45 items, evaluating three primary dimensions: 1) symptom distress, 2) interpersonal relationships, and 3) social role performance. While the total score range is 0–180, the cutoff score between clinical and nonclinical populations is 63 [15]. This scale is broadly used, showing good validity, internal consistency (0.93), and test–retest reliability (*r* = 0.84) [16]. Moreover, studies among clinical outpatients have also found it to be sensitive to change [17]. The alpha coefficient of the OQ-45 in the current sample indicated high internal reliability (Cronbach’s alpha = 0.95).

Psychological well-being scale (PWB) [18]. A self-report questionnaire designed to evaluate six elements of psychological well-being: personal growth, purpose in life, self-acceptance, environmental mastery, positive relations with others, and autonomy. Participants are requested to respond on a 6-point Likert scale ranging from 1 (strongly disagree) to 6 (strongly agree), and a mean score is calculated for each dimension of well-being, with higher scores indicating higher well-being in all domains. Previous studies among psychiatric outpatients have shown that this scale is sensitive to changes in well-being [19]. For the purpose of the current study, we utilized the total score of the PWB, as well as the personal growth, purpose-in-life, and self-acceptance factors. The alpha coefficient of the PWB in the current sample indicated high internal reliability (Cronbach’s alpha = 0.95).

Fear of COVID-19 (FCV-19S) [20]. A self-report scale designed to measure fear of COVID-19. The questionnaire consists of 7 items describing pandemic-related emotional fear reactions. Items are rated on a five-item Likert-type scale ranging from 1 (strongly disagree) to 5 (strongly agree), and a total sum score is calculated. The total scale range is 7–35, with higher scores demonstrating higher fear of COVID-19. The scale recently showed good psychometric properties in an Israeli sample [21]. The alpha coefficient of the FCV-19S in the current sample indicated high internal reliability (Cronbach’s alpha = 0.92).

### Statistical Analysis

All demographic and pandemic-related variables were described by mean and standard deviation scores for continuous variables, and prevalence and percentages for dichotomous variables. Associations between outcome variables and the different factors of the measures were reported using Pearson correlations, and p values were set at *p* < 0.05 or *p* < 0.01. Differences between baseline levels and post-lockdown measurements across all outcome variables were assessed using t-tests for paired samples. *p* values were adjusted to account for multiple comparisons using the Bonferroni correction (significance value set at *p* < 0.006). Prediction of deterioration in distress was based on change scores calculated as the difference between post-lockdown distress and pre-lockdown distress. In order to assess deterioration in distress, change scores were rescaled to reflect deterioration, where improvement or no change in distress was set to 0. All statistical analyses were performed using the Statistical Package for the Social Sciences (SPSS) for Windows v.25 (IBM Corp. Armonk, NY).

## Results

Clinical and demographic characteristics of the analyzed sample, as well as work and exposure characteristics during the pandemic, are presented in Table 1. Participants’ age ranged from 18 to 72, M = 37.11, SD = 13.53. The majority of the sample comprised female participants (69.9%). 60.3% of the sample were from a low socioeconomic status (SES), and 39.7% of the sample had a higher education. Of the total sample, 24 had been diagnosed with anxiety disorders (32.9%); 13 had been diagnosed with eating disorders (17.8%); 10 had been diagnosed with depression (13.7%); 10 had been diagnosed with adjustment disorders (13.7%); eight had been diagnosed with attention disorders (11.0%); three had been diagnosed with schizophrenia (4.1%); and five had been diagnosed as “Suspected mental and behavioral disorders” (6.8%). Most of the sample reported that they were working during the time of the pandemic (53.4%), with most of their children being at school during this period of time (70.6%). About nine percent (9.6%) reported that they had chronic background diseases. Eleven percent of the sample reported that they had family members who were still in isolation; 6.8% had a close friend infected; and 16.4% reported that they had a family member who had died of COVID-19.

**TABLE 1:** Demographic characteristics and current COVID-19 characteristics of the study sample (*n* = 73). Distress and well-being among psychiatric patients in the aftermath of the first COVID-19 lockdown in Israel: A longitudinal study, Israel, 2020.

Baseline demographic and clinical characteristics		Status during COVID-19 pandemic	
Age (M, sd)	37.11 (13.53)	Employment during lockdown	
Gender		Employed	39 (53.4%)
Male	22 (30.1%)	Unemployed	34 (46.6%)
Female	51 (69.9%)	Children at home during lockdown (*n* = 33)	
Country of birth		Yes	10 (29.4%)
Israel	56 (76.7%)	No	23 (70.6%)
Other	17 (23.3%)	Main caregiver during lockdown (*n* = 33)	
Marital status		Person or spouse	17 (51.5%)
Single	24 (32.9%)	Both parents	14 (42.4%)
Living with partner	9 (12.3%)	Babysitter/family members/others	2 (6.0%)
Married	34 (46.6%)	Background diseases	
Other	6 (8.2%)	No background diseases	66 (90.4%)
Socioeconomic status		Background diseases exist	7 (9.6%)
Below average	44 (60.3%)	Direct contact with COVID-19 patient	
Average	16 (21.9%)	Direct contact with patient	1 (1.4%)
Above average	13 (17.8%)	No direct contact	71 (97.3%)
Education		Family members in isolation	
High school	32 (43.8%)	Family member isolated	8 (11.0%)
Graduate studies	29 (39.7%)	No family member is isolated	64 (87.7%)
Post-graduate	12 (16.5%)	Patient is currently in isolation	
Main clinical diagnosis at baseline		Currently isolated	5 (6.8%)
Under observation	5 (6.8%)	Not isolated	67 (91.8%)
Schizophrenia	3 (4.1%)	Close person infected with COVID-19	
Depression	10 (13.7%)	Had a close friend infected	5 (6.8%)
Attention disorder	8 (11.0%)	No close friend was infected	67 (91.8%)
Adjustment disorder	10 (13.7%)	Family member dying of COVID-19	
Anxiety disorder	24 (32.9%)	COVID-19 death in family	12 (16.4%)
Eating disorder	13 (17.8%)	No COVID-19 death in family	60 (82.2%)

Table 2 presents the zero-order correlations of the main outcome measures with COVID-19 pandemic-related variables, and their associations with fear of COVID-19 (FCV-19S). As can be seen, fear of COVID-19 showed a significant positive correlation with all patient outcome scales (OQ-45), as well as an inverse correlation with psychological well-being (PWB) scales. An inverse correlation was also found between patient outcome scales (OQ-45) and psychological well-being (PWB) scales.

**TABLE 2:** Zero-order correlations of the main outcome variables during the COVID-19 pandemic, and their associations with fear of COVID-19 (FCV-19S) of the Total sample (*n* = 55). Distress and well-being among psychiatric patients in the aftermath of the first COVID-19 lockdown in Israel: A longitudinal study, Israel, 2020.

		1	2	3	4	5	6	7	8
1	Fear of COVID-19								
2	Symptomatic Distress	0.51**							
3	Interpersonal Distress	0.32**	0.77**						
4	Social Role	0.41**	0.73**	0.64**					
5	Overall Distress	0.49**	0.97**	0.86**	0.82**				
6	Personal Growth	−0.23	−0.35**	−0.40**	−0.25*	−0.37**			
7	Purpose In Life	−0.32**	−0.69**	−0.61**	−0.59**	−0.71**	0.51**		
8	Self-Acceptance	−0.28*	−0.76**	−0.74**	−0.61**	−0.79**	0.43**	0.72**	
9	General Well-Being	−0.40**	−0.81**	−0.78**	−0.66**	−0.84**	0.61**	0.84**	0.89**

Notes. Fear of COVID-19 = FCV-19S; Symptomatic Distress = Outcome Questionnaire-45, Symptom Distress scale; Interpersonal Distress = Outcome Questionnaire-45, Interpersonal Relationships scale; Social Role = Outcome Questionnaire-45, Social Role scale; Overall Distress = Outcome Questionnaire-45, Total scale; Personal Growth = Psychological Well-Being, Personal Growth scale; Purpose In Life = Psychological Well-Being, Purpose In Life scale; Self-Acceptance = Psychological Well-Being, Self-Acceptance scale; General Well-Being = Psychological Well-Being, Total scale.

Table 3 presents the means and standard deviations, and the level of significance of the differences of the main outcome variables at baseline, as compared with the same variables measured at the end of the lockdown. As can be seen, no statistically significant differences were observed in any of the symptom distress sub-scores (OQ-45) or in the total score. Nonetheless, significant differences in well-being were observed, t (55) = 3.11, *p* < 0.01, indicating a significant decrease in personal growth from baseline (M = 4.47, SD = 0.88) to the time of the pandemic (M = 4.16,SD = 0.89).

**TABLE 3:** Differences in main outcome variable before and after the COVID-19 pandemic. Distress and well-being among psychiatric patients in the aftermath of the first COVID-19 lockdown in Israel: A longitudinal study, Israel, 2020.

	Total sample (*n* = 55)		
	Baseline	During pandemic	*t*
Symptomatic Distress	41.13 (18.94)	43.40 (19.55)	−0.93
Interpersonal Distress	17.46 (6.37)	17.28 (7.64)	0.21
Social Role	12.10 (5.78)	11.78 (5.91)	0.39
Overall Distress	70.70 (28.81)	72.46 (30.63)	−0.47
Personal Growth	**4.47 (0.88)**	**4.16 (0.89)**	3.11***
Purpose In Life	3.99 (0.90)	3.85 (0.91)	1.49
Self-Acceptance	3.48 (1.13)	3.51 (1.25)	-0.18
General Well-Being	3.87 (0.79)	3.78 (0.83)	1.12

Notes. Symptomatic Distress = Outcome Questionnaire-45, Symptom Distress scale; Interpersonal Distress = Outcome Questionnaire-45, Interpersonal Relationships scale; Social Role = Outcome Questionnaire-45, Social Role scale; Overall Distress = Outcome Questionnaire-45, Total scale; Personal Growth = Psychological Well-Being, Personal Growth scale; Purpose In Life = Psychological Well-Being, Purpose In Life scale; Self-Acceptance = Psychological Well-Being, Self-Acceptance scale; General Well-Being = Psychological Well-Being, Total scale. p values were adjusted to account for multiple comparisons using the Bonferroni correction (significance value set on *p* < 0.006). Bold indicates significance of <.05.

An examination of changes in distress from baseline to post-pandemic indicated that 30 (54.4%) of the patients showed elevations in distress, while the other patients either exhibited no change, or improvement. Results of hierarchical linear regression assessing the predictive effects of demographic, clinical, and COVID-19 related factors on elevation in distress from pre-to post-pandemic are presented in Table 4. Model 1 and Model 2, containing age, gender, marital status, frequency of psychiatric ER visits, and total duration of psychiatric care did not significantly contribute to the prediction of elevation of distress, F (3,56) = 1.51, R^2^ change = 0.07, *p* = 0.22; F (2,54) = 0.52, R^2^ = 0.02, *p* = 0.52, respectively. However, Model 3 produced a significant effect which explained 8.9% of the variance, F (1,53) = 5.79, R^2^ change = 0.089, *p* < 0.05. In Model 1, age, gender, and marital status did not significantly predict deterioration. Furthermore, clinical characteristics of frequency of psychiatric ER visits and duration of total psychiatric care did not significantly predict deterioration. In Model 3, controlling for the abovementioned demographic and clinical characteristics, fear of COVID-19 significantly predicted deterioration (B = 0.84, t = 2.40. *p* < 0.05), indicating that higher fear of COVID-19 is associated with elevations in distress.

**TABLE 4:** Prediction of deterioration in distress by demographic, clinical, and COVID-19 related factors of the Total sample (*n* = 55). Distress and well-being among psychiatric patients in the aftermath of the first COVID-19 lockdown in Israel: A longitudinal study, Israel, 2020.

	Model 1				Model 2				Model 3				R^2^ change
	B	SE	Beta	t	B	SE	Beta	t	B	SE	Beta	t	
Demographic characteristics													
Age	−0.25	0.19	−0.18	−1.31	−0.23	0.19	−0.17	−1.22	−0.23	0.18	−0.16	−1.25	
Gender	−7.81	5.67	−0.18	−1.37	−7.06	5.74	−0.16	−1.22	−8.43	5.53	−0.19	−1.52	
Marital status	9.22	5.46	0.23	1.68	8.55	5.73	0.22	1.49	9.89	5.52	0.25	1.79	0.07
Clinical characteristics													
Frequency of psychiatric ER					2.23	2.23	0.10	0.78	1.86	2.14	0.11	0.86	
Duration of total psychiatric care					−0.00	0.002	−0.13	−0.95	−0.00	0.00	−0.13	−1.01	0.02
COVID-19 fear													
Fear of the COVID-19 pandemic									**0.84**	**0.352**	**0.30**	2.40*	0.08*

Notes. *p < 0.05. Bold indicates significance of <.05.

## Discussion

In this study we aimed to examine the psychological effects of the COVID-19 pandemic among psychiatric patients by comparing levels of distress and well-being before and after the lifting of the pandemic lockdown. The results of the study indicated that, overall, there was no significant increase in distress during the lockdown. Nonetheless, there was a significant decrease in the personal growth facet of well-being. Furthermore, the results indicated that 54.4% of the patients showed some deterioration in distress, and that this deterioration was positively and significantly associated with the fear of COVID-19, above and beyond demographic and clinical factors.

Several studies have previously reported that patients suffering from mental illnesses suffer from anxiety and depression during the pandemic. Nonetheless, most of these studies employed a cross-sectional design rather than a longitudinal one [22–25]. This differential methodological design is likely to affect the pattern of results. Another possible explanation to account for the differential pattern of results is the use of specific measures for anxiety, depression, and well-being, which could have also affected the pattern of results. Additional studies are needed to further explore whether sample characteristics or the selection of measures influence the patterns of findings. These studies should focus on the ongoing effects of the pandemic and lockdowns across various instruments and larger and diverse samples.

Although several studies have found elevated levels of distress in psychiatric patients during the pandemic, there have also been some reports indicating minimal adverse mental health effects in different populations. To illustrate, Zhang and Ma [26] assessed 263 respondents in China and found that the pandemic was associated with only a mild stressful impact. Fried et al. [27] followed students during the pandemic outbreak and found that students actually reported a slight improvement in mental health problems, as well as decreases in COVID-19 related concerns. Looking at other significant national events, Bystritsky et al. [28] assessed the response of 41 patients with OCD or panic disorders to a major earthquake in Northridge, California, and found no significant exacerbation of their primary symptoms following the earthquake, compared to baseline measurements. These findings suggest that the effect of the pandemic might be more apparent in the long run.

Patients reported a decrease in well-being in the aftermath of the lockdown in Israel. One potential explanation to account for the effect of the pandemic on patients’ well-being is the development of economic uncertainty during the lockdown, which might have dramatically affected the lives of patients coming from a low socioeconomic background and treated in public mental health facilities. The results of our study indicate that the decrease in well-being is primarily manifested in the sense of personal growth, which pertains to the sense of continued growth and development as a person, as well as openness to new experiences [29]. Studies indicate that personal growth is negatively affected by stressful situations [30], and positively associated with social and personal activities such as physical activity [31], leisure activities [32], and meaningful social interactions [33]. These domains were all subjected to a complete cessation during the pandemic quarantine.

Finally, the results of our study indicated that fear of COVID-19 predicted a deterioration in overall distress above and beyond the effect of demographic and clinical factors. Cross-sectional studies performed in normative populations have previously indicated that fear of COVID-19 is positively associated with anxiety, stress, and depression [21], exhaustion, loneliness, nervousness, and anger [34], and negatively associated with mental well-being [35]. The results of the current study further indicate that fear of COVID-19 not only co-occurs with distress, but can also predict the longitudinal course of deterioration, as manifested in an increase of overall distress compared to baseline measurement. Previous studies assessing emotional consequences to a stressful event have indicated that patients can develop symptoms that are directly associated with the adverse event. For example, Shasha et al. [36] found that 35.4% of the patients suffering from panic attacks also demonstrated posttraumatic stress symptoms in reference to the attacks. Thus, one potential explanation to account for the current findings is that the increase in distress found in a significant percentage of our sample is specifically associated with the outbreak of the pandemic, and the fear of its consequences. Such a potential explanation should be explored in future studies.

Several limitations should also be noted. As the time gap between measurements was relatively large, other causal explanations may explain the observed changes in measures of distress and well-being. Additional studies should be performed to control for potential confounding factors. Moreover, although gender was not a predictive factor of deterioration in symptom distress, this lack of effect may be due to the small number of participants, which did not allow for further examination of the effects of the pandemic for female and male participants. Future studies should aim to explore whether the pandemic may have a differential effect on mental illness progression across female and males. Another potential limitation is the use of specific measures, which were predetermined by the purposes of the previous study. Although we used the broadest selection of instruments available from the previous study to mitigate this limitation, future studies should evaluate their sensitivity by utilizing other symptom and well-being scales. Since the study was based on a previously recruited sample, potential selection bias cannot be ruled out. Additional studies should be performed so as to assess the external validity of our findings. Finally, as this study was conducted among a group of patients treated in a psychiatric hospital in Israel, additional studies are needed to further explore the generalizability of our findings across different cultures and clinical settings. Notwithstanding these limitations, our findings provide initial results indicating changes in well-being among psychiatric patients and provide the grounds for future investigations of the short- and long-term effects of the pandemic on psychiatric patients’ mental health.

### Conclusion

The results of the current study indicate that there were no significant increases in distress during the lockdown, but that personal growth facet of well-being significantly decreased. Furthermore, increases in distress were associated with the fear of COVID-19, above and beyond demographic and clinical factors. These findings have several important implications. The lack of a significant difference in overall level of distress before and after the pandemic, and specifically upon the lifting of the lockdown, might suggest that the pandemic had relatively low short-term effects on psychiatric patients’ mental health. However, the significant decrease in well-being, as well as the increase in distress among 54.4% of the sample, might suggest that the mental consequences of the pandemic may be more apparent in the long run. Future initiatives assessing the impact of the pandemic should therefore focus on exploring both the short- and long-term effects of the pandemic on this vulnerable population. Furthermore, the predictive effect of the fear of COVID-19 on distress elevation, beyond clinical and demographic factors, may suggest that fear reactions associated with the pandemic might be a facilitator of negative changes in patients’ mental health. Additional longitudinal studies employing larger samples are needed in order to validate these findings, so as to determine whether interventions aimed at relieving COVID-19-related fears and anxieties might be beneficial for patients.
